# Gender-Based Disparities in Rural Versus Urban Patients Undergoing Cardiac Procedures

**DOI:** 10.7759/cureus.16672

**Published:** 2021-07-27

**Authors:** Vinila S Baljepally, David C Wilson

**Affiliations:** 1 Medicine, Health and Society, Vanderbilt University, Nashville, USA; 2 Internal Medicine, University of Tennessee Medical Center, Knoxville, USA

**Keywords:** gender-based differences, healthcare outcomes, urban / rural patients, urban and rural community, health care disparity

## Abstract

Introduction

Rural populations have higher rates of diabetes and hypertension (HTN) with disparities in outcomes among patients presenting to the emergency room with heart attack and stroke. However, it is unclear whether there are any sex differences among patients presenting for cardiac procedures from rural versus urban areas. Our study aimed to investigate gender-based differences in baseline characteristics and procedural outcomes among rural and urban residents presenting for cardiac catheterization and percutaneous interventional procedures.

Methods

We assessed baseline conditions and outcomes in 1775 patients who underwent cardiac catheterization and or Percutaneous Coronary Intervention at the University of Tennessee Medical Center between July 2018 to October 2019 from rural as well as urban areas. Baseline conditions assessed were diabetes, HTN, stroke, peripheral vascular disease, heart failure, and prior bypass surgery. Outcomes assessed were vascular/bleeding complications, duration of the procedure, and mortality.

Results

There were significant gender-based inter-group differences in outcomes between rural versus urban residents. In general, both rural and urban males had significantly longer procedure times and higher mortality than rural or urban females (P=0.01). Among females, rural women had longer procedure times than urban women. Bleeding complications were greater among rural residents than urban residents (p≤0.001), with rural females having the highest bleeding complication rate. Mortality was also higher among rural females compared to their urban counterparts (p=0.01).

Significant gender-based inter-group differences were noted between rural versus urban residents. While the incidence of stroke was higher among rural and urban females compared to males, the peripheral vascular disease was more common among males. The history of coronary artery bypass graft (CABG) was more commonly seen among rural males than females. Rural and urban males had significantly longer procedure times than females, particularly urban females (P=0.01). Among women, rural females had longer procedure times, higher vascular/bleeding complications, and greater mortality than urban females. Mortality was higher among rural men and women compared to urban men or women (p=0.01). Rural women had the highest bleeding/vascular complications.

Conclusions

We found significant gender-based differences between rural versus urban patients. While rural females had a higher incidence of stroke, peripheral vascular disease and a history of CABG were more commonly seen among rural males. Overall, rural males had higher mortality than females (P=0.01). Among women, rural females had longer procedure times, higher bleeding complications, and greater mortality than urban females. Being aware of such gender-based differences may help physicians take steps to improve outcomes. Information derived from our study may also be useful for policymakers in directing healthcare funding to lower gaps in the care of patients such as those with peripheral vascular disease, ultimately leading to better health outcomes.

## Introduction

Studies have shown sex differences in the utilization of cardiac interventional procedures that favor men [1]. Nguyen et al. found that women are subjected to cardiac catheterization and are placed on guideline-directed medical therapy less often than men after an acute myocardial infarction [2]. Zhao et al. found sex differences in medication prescription in primary care [3]. Women admitted to hospitals in a region of northern Italy with a diagnosis of cardiovascular disease were treated differently, which could not be explained by age or severity of disease [4]. One study found that men were nearly 2.5 times more likely to have interventional procedures compared to women [5]. Similar to disparities based on gender, there are known differences in the care of rural versus urban residents [6]. Rural residents have a higher prevalence of diabetes and coronary heart disease as suggested by the cross-sectional study by O’Connor and Wellenius of more than 214,000 respondents using data from the US centers for disease control and prevention [7]. There are significant challenges faced by rural residents in access to care and mortality was higher for rural residents in a large cohort study of low-income Medicare beneficiaries dually enrolled in Medicaid [8,9]. While there are some known disparities based on gender and rural residence for patients presenting with acute myocardial infarction and heart failure, it is unclear whether there are any sex differences among patients presenting for cardiac catheterization and percutaneous interventional procedures from rural and urban areas.

The aim of our study was to investigate gender-based differences in baseline characteristics and procedural outcomes among rural and urban residents presenting for cardiac catheterization and percutaneous coronary interventional procedures.

## Materials and methods

Study design

Our study is a retrospective, non-randomized, single-center study of patients (n=1775) who presented to the University of Tennessee Medical Center between July 2018 to October 2019 from rural and urban areas. Given the retrospective chart review nature of the study, permission was given by the University of Tennessee Medical Center’s Institutional Review Board and the requirement for patient consent was waived.

Study patients and inclusion/exclusion criteria

We included patients who presented to the cardiac catheterization lab either directly or via the emergency room with complaints of chest pain and stable angina, unstable angina, and ST-elevation myocardial infarction (STEMI) were included. We excluded patients when an address was unclear or could not be verified. We excluded patients from zip codes/counties that had a population between 5000 and 20,000. 

Data gathering and statistical analysis

Factors Assessed

Demographics, clinical history, and angiographic data were collected for each patient by chart review. We assessed the following baseline conditions: diabetes, hypertension (HTN), stroke, peripheral vascular disease, prior coronary artery bypass graft (CABG) surgery, and congestive heart failure (CHF). The American Heart Association (AHA) criteria were utilized for the definition of STEMI by EKG and similar AHA criteria were utilized for the diagnosis of unstable angina and non-ST-elevation myocardial infarction (NSTEMI) based on symptoms and troponin elevations. Association between baseline conditions and gender as well as rural versus urban residence was evaluated and compared between groups. Between-group differences in age, gender, above-mentioned baseline conditions, body mass index (BMI), procedure time, and vascular complications including bleeding complications were assessed (Table 1).

**Table 1:** Baseline characteristics of patients presenting from rural and urban areas Variables are expressed as number (%) or mean ± standard deviation. P < 0.05 indicates the difference between the groups is statistically significant. TIA: transient ischemic attack, CABG: coronary artery bypass grafting, BMI: basic metabolic index, UA: unstable angina, NSTEMI: non-ST elevation myocardial infarction, STEMI: ST-elevation myocardial infarction.

Characteristics	Urban (N = 1416)	Rural (N = 359)	P-value
Age (years)	64.87 ± 11.62	64.86 ± 11.17	0.97
Sex			
Male	900 (63.56%)	221 (61.56%)	0.48
Female	516 (36.44%)	138 (38.44%)	0.48
Clinical history			
Diabetes mellitus	491 (34.67%)	124 (34.54%)	0.96
Hypertension	1146 (80.93%)	289 (80.5%)	0.85
Congestive heart failure	205 (14.47%)	57 (15.87%)	0.50
Stroke	130 (9.18%)	38 (10.58%)	0.42
Peripheral vascular disease	563 (39.75%)	210 (58.5%)	<0.001
CABG	35 (2.47%)	33 (9.19%)	<0.001
BMI (kg/m^2^)	30.9 ± 6.1	29.8 ± 5.8	0.87
Clinical presentation			
Stable angina	653 (46.11%)	164 (45.68%)	0.88
UA/NSTEMI	425 (30.01%)	116 (32.31%)	0.39
STEMI	69 (4.87%)	15 (4.17%)	0.58
Procedure time (min)	12.92 ± 15.05	13.7 ± 10.55	0.46

Rural/Urban Definition

For the purposes of this study, we considered "rural residents" as those patients who live in counties with a population of less than 5000. We utilized Appalachian Regional Commission (ARC) maps and Zipcode information to classify an area as rural. A county was classified as "urban" if the population was >20,000. Most non-rural counties in our study had a population of more than 20,000. We excluded patients from counties that had a population between 5000 and 20,000.

Statistical analysis

The normal distribution of variables was assessed with the Shapiro-Wilk normality test. Continuous variables were expressed as means±standard deviation and categorical variables were expressed as proportions and percentages. Continuous variables were analyzed with a one-way ANOVA test and categorical variables were analyzed using Fisher’s exact test. A p-value of <0.05 indicated statistical significance. Statistical analysis was performed using GraphPad Prism, version 9.

## Results

Of the studied population, 20.2% (n= 359) were rural residents; 38.4% of rural patients (n=138) were female. No significant between-group differences were noted in baseline factors of age and BMI. Also, there were no significant differences noted by the type of presentation such as chest pain, unstable angina NSTEMI, or STEMI. Inter-group gender-based differences are as described below.

Rural-urban sex differences in baseline conditions

**Figure 1:**
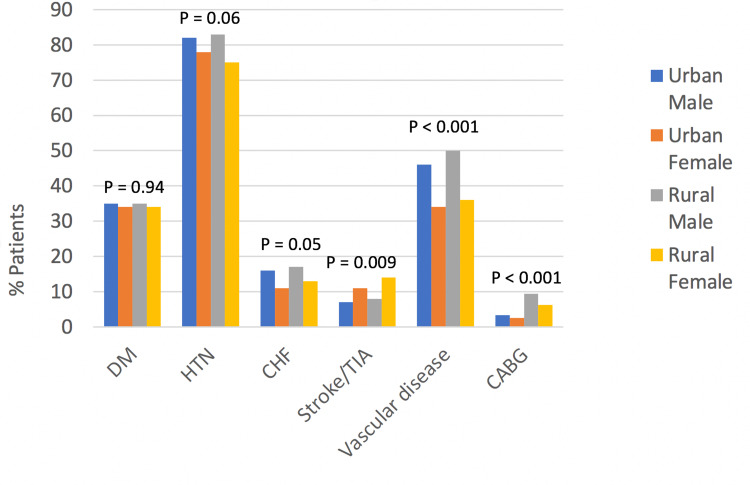
Association between rural-urban gender and baseline clinical factors DM: diabetes mellitus, HTN: hypertension, CHF: congestive heart failure, TIA: transient ischemic attack, CABG: coronary artery bypass graft surgery.

No significant sex differences were noted between rural and urban residents in the incidence of Diabetes (Figure 1, p = 0.94). Although not statistically significant, there was a trend toward a higher incidence of HTN and CHF among males compared to females. The trend was more in rural men than in urban men.

Statistically significant differences were however noted in the incidence of stroke, peripheral vascular disease, and CABG with interesting trends. Male and female rural residents had a higher incidence of stroke compared to urban residents of the same gender. Stroke incidence was greater in females than males and more common among rural females than urban females (Figure 1). Peripheral vascular disease and history of CABG were both more common in rural males than rural or urban females (Figure 1).

Rural-urban sex differences in outcomes

Significant rural-urban gender-based differences were also noted in outcomes. In general, rural and urban males had significantly longer procedure times than urban females (P=0.01). Among women, rural females had longer procedure times (P=0.01), higher vascular/bleeding complications, and greater mortality than urban females (P=0.03).

**Figure 2:**
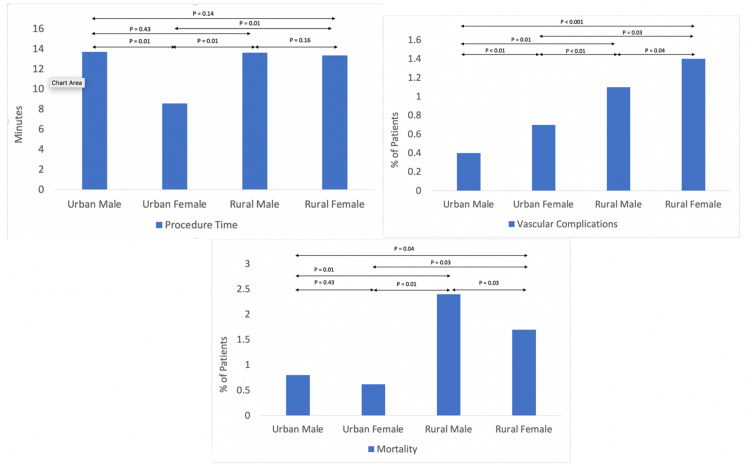
Rural-urban gender-based differences in outcomes Rural-urban gender-based differences in procedure times (top left), vascular/bleeding complications (top right), and mortality (bottom).

Bleeding/vascular complications and mortality were both higher among rural males and females compared to urban males or females (Figure 2). Rural women had the highest bleeding/vascular complications compared to urban women or rural/urban men (Figure 2).

## Discussion

Coronary artery disease remains the predominant cause of death among women in developed countries and several studies from the USA and other countries have shown that compared to men with cardiovascular disease, women are less likely to receive interventional cardiac procedures [4]. Nante et al. found that women admitted to hospitals in the Piedmont region of northern Italy with a diagnosis of cardiovascular disease were treated with percutaneous intervention or surgery significantly less often than men and this could not be explained by the age or severity of the disease [4]. Chiriboga et al. found that men were nearly 2.5 times more likely to have interventional procedures compared to women [5]. Similar to disparities in gender, there are known differences in the care of rural versus urban residents [6]. Increased incidence of diabetes and heart disease among rural residents and disparities in access to basic healthcare are previously known [7]. A North Carolina rural health report found that during the past decade, access to healthcare services provided by rural hospitals has changed in two major ways: (1) there has been a substantial increase in the number of rural hospitals that have closed, and (2) many rural hospitals have reduced or terminated services historically considered to be essential hospital services. These trends between 2009 and 2017 have resulted in worsening disparities between rural and urban hospitals [8]. Differences in mortality based on poverty level and rural residence, sex and race differences in outcomes in young adult patients with cardiovascular disease risk factors, and sex disparities in the outcome of stroke patients have been reported [9-11]. One study from Spain showed that compared to men, women had a lower level of knowledge as well as the perception of stroke risk factors and warnings [12].

However, studies showing rural-urban gender-based differences in baseline health conditions and outcomes among patients in the United States presenting primarily for cardiac catheterization and coronary interventional procedures are lacking. Studies have not evaluated the effect of both gender and rural residence in evaluating coronary invasive procedural outcomes in the United States. We assessed if any sex differences exist between rural versus urban residents presenting for such procedures. Our study showed significant sex differences among rural and urban populations. Although the patient population did not significantly differ in age, sex, BMI, or clinical presentation, statistical differences were noted between males and females from rural versus urban areas.

Significant differences were noted in the incidence of stroke, peripheral vascular disease, and prior CABG. While the incidence of stroke was higher among rural and urban females compared to males, the peripheral vascular disease was more common among rural and urban males. The history of CABG was more commonly seen among rural males than females.

Significant rural-urban gender-based differences were also noted in outcomes. In general, rural and urban males had significantly longer procedure times than urban females (P=0.01). Among women, rural females had longer procedure times (P=0.01), higher vascular/bleeding complications, and greater mortality than urban females (P=0.03).

Bleeding/vascular complications were higher among rural men and women compared to urban men or women (p= 0.01, Figure 2). Mortality was higher among rural men and women compared to urban men or women. Rural women had the highest bleeding/vascular complications (Figure 2).

We hypothesize that the higher mortality among rural compared to urban patients is at least in part to the higher rates of vascular disease and history of CABG which were both higher among rural residents. Similarly, higher vascular/bleeding complications and prior CABG may have led to greater mortality among rural women compared to their corresponding urban counterparts. Understanding gender-based differences, as suggested by our study, may help to improve the quality of healthcare delivered to high-risk groups such as rural residents and subsequently lead to better outcomes. Information derived from our study may help policymakers enhance funding to increase healthcare access for rural patients, eventually lowering the gaps in the treatment of modifiable risk factors such as diabetes and vascular disease.

The main limitations of our study include its retrospective, single-center design and the smaller sample size of patients from rural areas.

## Conclusions

Our study suggests gender-based disparities among patients presenting for cardiac procedures from rural versus urban areas. While the incidence of stroke was higher among rural females, peripheral vascular disease and a history of CABG were more commonly seen among rural males. Despite longer procedure times in men compared with women, rural women had the highest bleeding/vascular complications. Mortality was higher among rural men and women compared to urban men or women. Among women, rural females had greater mortality than urban females. Such differences are likely due to ongoing challenges in access to quality healthcare and management of modifiable risk factors. By highlighting the existence of gender-based disparities, our study may ultimately help in improving outcomes, especially for rural patients.
